# Differential Expression of S100A8 in Tumor and Immune Compartments of Endometrial Carcinoma and Its Clinical Relevance

**DOI:** 10.3390/medicina61111918

**Published:** 2025-10-25

**Authors:** Dae Hyun Song, Min Hye Kim, Juseok Yang, Hyen Chul Jo, Ji Eun Park, Jong Chul Baek

**Affiliations:** 1 Department of Pathology, School of Medicine, Gyeongsang National University, Jinju 52727, Republic of Korea; golgy@hanmail.net (D.H.S.); joymine86@naver.com (M.H.K.); 2 Department of Pathology, Gyeongsang National University Changwon Hospital, Changwon 51472, Republic of Korea; 3 Institute of Health Science, Gyeongsang National University, Jinju 52727, Republic of Korea; yangandshin@gmail.com (J.Y.); 73hccho@gnuh.co.kr (H.C.J.); obgy@gnu.ac.kr (J.E.P.); 4 Department of Pathology, Gyeongsang National University Hospital, Jinju 52727, Republic of Korea; 5 Department of Obstetrics and Gynecology, School of Medicine, Gyeongsang National University, Jinju 52828, Republic of Korea; 6 Department of Obstetrics and Gynecology, Gyeongsang National University Changwon Hospital, Changwon 51472, Republic of Korea

**Keywords:** endometrial cancer, S100A8, tumor microenvironment, immune cells, prognostic factors

## Abstract

*Background and Objectives*: S100A8 regulates inflammatory responses and immune cell activation and is overexpressed in several solid tumors. However, its clinicopathological significance in endometrial carcinoma (EC) remains unclear. This study aimed to evaluate the expression patterns of S100A8 in both tumor and immune cells of EC and examine its association with clinicopathological features. *Materials and Methods*: Fifty-two formalin-fixed, paraffin-embedded EC specimens were analyzed using tissue microarray-based immunohistochemistry. S100A8 expression was assessed in tumor and immune cells. The tumor proportion score (TPS), tumor staining intensity (TI), and immune proportion score (IPS) were dichotomized into low and high categories (TPS/IPS: ≤30% vs. ≥31%; TI: 0–1+ vs. 2–3+). Correlations with clinicopathological parameters were examined using the chi-square and Fisher’s exact tests. *Results*: A low TPS, high TI, and high IPS were observed in 51.9%, 63.5%, and 57.7% of patients, respectively. TPS and TI showed no significant correlation with clinicopathological variables, including age, tumor size, invasion depth, histologic grade, T stage, and N stage (all *p* > 0.05). By contrast, IPS was significantly associated with patients’ age (*p* = 0.044) and histologic grade (*p* = 0.012), with older patients and those with higher-grade tumors demonstrating a higher IPS. A positive correlation was observed between TPS and IPS (*p* = 0.044), whereas TI did not correlate with IPS (*p* = 0.253). *Conclusions*: S100A8 expression in immune cells, but not in tumor cells, is associated with age and tumor grade in EC. Therefore, immune-related S100A8 expression may serve as a biomarker of the tumor immune microenvironment, warranting further investigation into its prognostic and therapeutic implications.

## 1. Introduction

Endometrial carcinoma (EC) is one of the most common malignancies of the female reproductive system, with its global incidence increasing 2.55-fold over the past three decades and surpassing 420,000 new cases in 2022 [1]. In Western countries, the incidence of endometrial carcinoma is steadily increasing, with not only endometrioid but also aggressive non-endometrioid subtypes showing a marked rise in recent decades. In Korea, the incidence of EC has risen across all histological subtypes, mirroring global trends observed in developed nations. Although notable survival improvements have been observed primarily in endometrioid carcinoma, outcomes for serous and clear cell subtypes have remained largely unchanged [2,3]. Even within endometrioid EC, prognosis is heterogeneous, with poor outcomes associated with high histologic grade, advanced stage, lymphovascular space invasion, and molecular alterations such as p53 mutations [4,5,6]. This clinical heterogeneity underscores the urgent need for reliable biomarkers to facilitate early diagnosis, risk stratification, and individualized therapeutic strategies.

The S100 protein family—first identified in the bovine brain tissue in 1965 [7]—comprises more than 20 members characterized by conserved EF-hand calcium-binding domains, yet each member exhibits distinct biological functions. These small proteins are predominantly clustered on chromosome 1q21 [8]. While S100 proteins share structural similarities, they perform diverse cellular functions, including the regulation of proliferation, differentiation, cytoskeletal dynamics, and inflammatory signaling [9,10]. Dysregulation of S100 proteins is frequently observed in various malignancies, where they contribute to tumor progression and modulation of the tumor immune microenvironment [9,10,11]. In particular, S100A8—which is expressed in both myeloid-lineage immune cells and tumor epithelial cells—has emerged as a critical mediator of tumor–immune interactions. S100A8 promotes the recruitment and accumulation of myeloid-derived suppressor cells (MDSCs), activates NF-κB and MAPK signaling, and facilitates tumor proliferation, invasion, and pre-metastatic niche formation [12,13,14]. Overexpression of S100A8 has been associated with poor survival in several solid tumors, including breast, bladder, gastric, lung, and liver cancers [15,16,17,18,19,20].

In EC, multiple S100 proteins, including S100A1, S100A2, S100A4, S100A8, and S100A9, have been implicated in tumor proliferation, invasion, epithelial–mesenchymal transition (EMT), and response to chemotherapy [11,21,22,23]. Functional studies suggest that S100A8 modulates tumor growth and sensitivity to chemotherapy [11], while its expression in immune cells may shape the tumor microenvironment and correlate with clinical outcomes. Despite these findings, the clinical and prognostic significance of S100A8 in EC—particularly regarding its differential expression in tumor versus immune compartments—remains poorly understood. To date, no study has comprehensively evaluated S100A8 expression in both compartments using tissue microarrays (TMAs).

Therefore, this study employed TMAs to comprehensively assess S100A8 expression in both tumor and immune cells and to investigate its associations with clinicopathological parameters. By clarifying whether tumor-intrinsic or immune-related S100A8 expression carries greater clinical relevance, we aimed to explore the potential of S100A8 as a prognostic biomarker in EC.

## 2. Material and Methods

### 2.1. Sample Collection

The tissue specimens used in this study were obtained from archived samples at the Department of Pathology, Gyeongsang National University Hospital, Jinju City, Republic of Korea, spanning an 8-year period from January 2002 to December 2009. The study cohort comprised 52 formalin-fixed, paraffin-embedded tissue specimens obtained from patients with histologically confirmed endometrioid endometrial cancer. All participants underwent comprehensive surgical management performed by a specialized gynecological oncologist, including total hysterectomy with bilateral salpingo-oophorectomy, systematic pelvic and para-aortic lymph node dissection, and omentectomy. A standardized surgical protocol was applied to ensure consistency in specimen collection and pathological evaluation. Diagnostic confirmation was performed independently by two qualified pathologists. Tumor staging and histological classification were determined according to the 2009 International Federation of Gynecology and Obstetrics (FIGO) guidelines and the tumor–node–metastasis classification system developed by the Union for International Cancer Control [24,25], respectively. Clinical data, including demographic characteristics and comprehensive pathological parameters, were retrospectively collected through a review of electronic medical records and systematically extracted and analyzed to establish clinicopathological correlations relevant to the study objectives.

### 2.2. TMA Construction and Ethical Approval

Surgically resected specimens were fixed overnight in 20% buffered neutral formalin and subsequently embedded in paraffin. Hematoxylin and eosin-stained slides were prepared from paraffin-embedded tissue blocks. Representative sections were reviewed, and the two most characteristic tumor areas were selected and marked. From each selected area, 3 mm tissue cores were obtained and transplanted into recipient TMA blocks.

This study was approved by the Institutional Review Board of Gyeongsang National University Hospital (GNUH; IRB No. 2020-04-006). The requirement for informed consent was waived owing to the retrospective nature of the study and the use of anonymized archival specimens. All research procedures were performed in accordance with the ethical principles outlined in the Declaration of Helsinki.

### 2.3. Immunohistochemical (IHC) Staining and Evaluation

**Figure 1:**
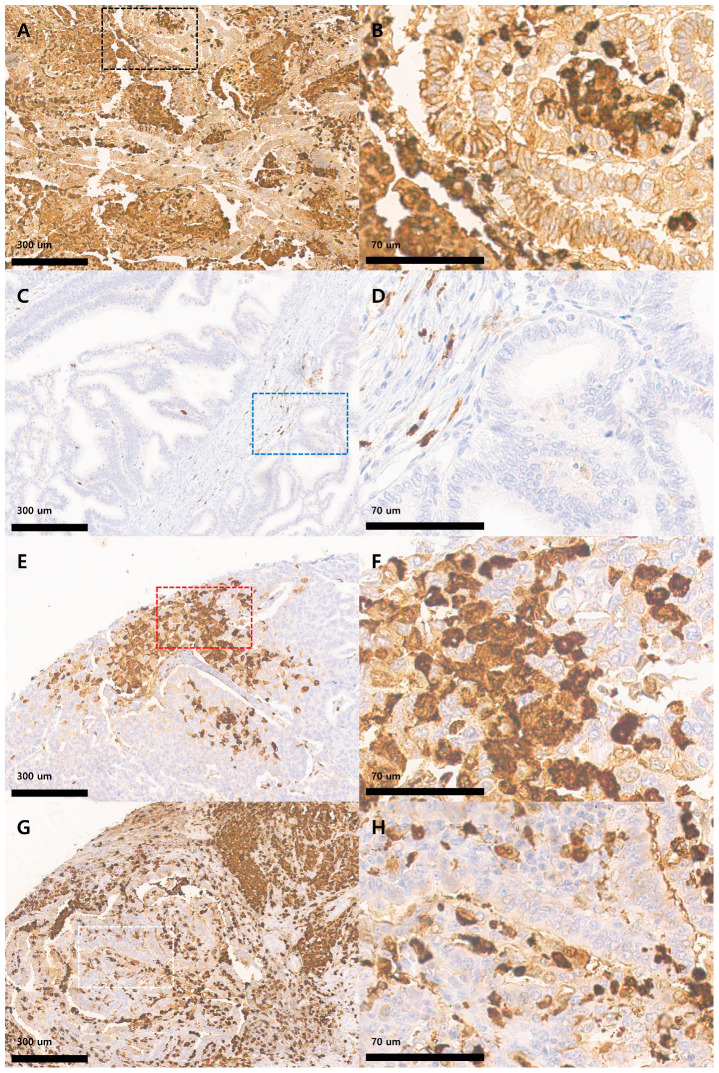
Expression of S100A8 in endometrioid endometrial carcinoma. (**A**) Tumor showing high tumor proportion (high TPS) and high tumor intensity (high TI) of S100A8 expression, with abundant S100A8-positive immune cells (high IPS) (×100). (**B**) Magnified view of the boxed area in (**A**) (black dotted rectangle, ×400). (**C**) Tumor exhibiting low TPS, low TI, and low IPS, indicating minimal S100A8 expression in both tumor and immune cells (×100). (**D**) Magnified view of the boxed area in (**C**) (blue dotted rectangle, ×400). (**E**) Tumor demonstrating high TPS and high TI but low IPS, reflecting strong S100A8 expression in tumor cells with minimal immune cell staining (×100). (**F**) Magnified view of the boxed area in (**E**) (red dotted rectangle, ×400). (**G**) Tumor displaying low TPS and low TI but high IPS, showing limited S100A8 staining in tumor cells but strong positivity in surrounding immune cells (×100). (**H**) Magnified view of the boxed area in (**G**) (white dotted rectangle, ×400).

IHC staining was performed on 4-μm thick sections prepared from TMA paraffin blocks using an automated stainer (Benchmark Ultra version 12.5; Ventana Medical Systems Inc., Tucson, AZ, USA) with a monoclonal anti-S100A8 antibody at 1:500 dilution (EPR3554; Abcam, Cambridge, UK). All slides were independently evaluated by two pathologists blinded to clinicopathological data, and discrepancies were resolved by consensus. Tumor proportion and immune proportion were assessed via a semi-quantitative analysis of S100A8 immunostaining in EC specimens. The tumor proportion score (TPS) was calculated as the percentage of viable tumor cells demonstrating cytoplasmic and/or membranous S100A8 immunoreactivity, whereas the immune proportion score (IPS) was defined as the percentage of S100A8-positive immune cells within the stromal compartment. Both the TPS and IPS were dichotomized at a 30% cutoff: cases with ≤30% positive tumor cells were classified as low TPS, and those with ≤30% positive immune cells were classified as low IPS. The tumor staining intensity (TI) of S100A8 was evaluated using a semi-quantitative four-tier grading system: 0 (negative), indicating the absence of staining or background-level reactivity; 1+ (weak positive), defined by faint cytoplasmic and/or membranous staining visible only at high magnification (×40); 2+ (moderate positive), characterized by clear staining at intermediate magnification (×20); and 3+ (strong positive), representing intense staining readily apparent at low magnification (×10). For analytical purposes, tumors exhibiting 0 or 1+ staining intensity were designated as having low TI, whereas those with 2+ or 3+ staining intensity were classified as having high TI. Figure 1 presents representative immunohistochemical images demonstrating four distinct S100A8 expression patterns in endometrioid endometrial carcinoma: co-expression in both tumor and immune cells (A, B), negative expression in both compartments (C, D), tumor-dominant expression (E, F), and immune cell-dominant expression (G, H).

### 2.4. Statistical Analysis

Associations between S100A8 expression and clinicopathological variables, including patients’ age, tumor size, invasion depth, histologic grade, T stage, and N stage, were evaluated using Pearson’s chi-square test and Fisher’s exact test, as appropriate. For analysis, each S100A8 parameter was dichotomized into low versus high groups (e.g., tumor proportion [low TPS: 0–30% vs. high TPS: 31–100%], tumor intensity [low TI: negative to weak vs. high TI: moderate to severe], and immune proportion [low IPS: 0–30% vs. high IPS: 31–100%]). The relationship between S100A8 expression in tumors and immune cells was also assessed using Pearson’s chi-square test. A two-sided *p*-value < 0.05 was considered statistically significant. All statistical analyses were performed using SPSS software (version 24.0; IBM Corp., Armonk, NY, USA).

The authors acknowledge the assistance of artificial intelligence-based language tools (ChatGPT, version GPT-5; OpenAI, San Francisco, CA, USA) in improving the clarity of English expression and in standardizing reference formatting. The authors have reviewed and edited the output and take full responsibility for the content of this publication.

## 3. Results

### 3.1. Patient Characteristics

A total of 52 patients with endometrioid EC were included in this study. Table 1 summarizes the clinicopathological characteristics of the cohort, along with the distribution and intensity of S100A8 expression in tumor and immune cells. Patients’ age ranged from 35 to 78 years, with a median of 51 years. The median tumor size was 3.5 cm (range, 0.7–10 cm), and the median depth of myometrial invasion was 0.3 cm (range, 0.01–4 cm). Most patients were diagnosed with uterine-confined, early-stage disease (stage I and II), whereas only two patients (3.8%) presented with stage III disease. Consistently, lymph node metastasis was rare, with 47 patients (90.4%) classified as N0 and only 5 (9.6%) showing nodal involvement (N1–N2). Histological grading according to FIGO revealed that 35 patients (67.3%) had lower-grade tumors (G1), whereas 17 (32.7%) had higher-grade tumors (G2 and G3).

### 3.2. IHC Patterns of S100A8 Expression

IHC analysis of S100A8 expression demonstrated heterogeneous patterns across tumor and immune compartments. The TPS for S100A8 expression was evaluated and stratified into three categories: 0–30%, 31–60%, and 61–100%. Overall, 51.9% of patients exhibited a TPS of 0–30% (low expression), 30.8% exhibited a TPS of 31–60%, and 17.3% demonstrated a TPS of 61–100%. For subsequent analyses, the TPS was dichotomized into two groups: low TPS (0–30%) and high TPS (31–100%). Based on this classification, 27 patients (51.9%) were assigned to the low TPS group and 25 (48.1%) were assigned to the high TPS group. TI analysis for S100A8 expression revealed a heterogeneous distribution in the study cohort. Six patients (11.5%) demonstrated a complete absence of expression (negative), whereas 13 (25.0%) demonstrated weak staining intensity. Moderate staining was the most frequently observed category, accounting for 21 patients (40.4%), followed by severe staining in 12 patients (23.1%). For further statistical evaluation, TI was dichotomized into TI-low (encompassing negative and weak staining intensity categories) and TI-high (comprising moderate and severe categories). Based on this classification, 19 patients (36.5%) were assigned to the TI-low group, whereas 33 (63.5%) were assigned to the TI-high group. The IPS was distributed as follows: 22 patients (42.3%) demonstrated an IPS of 0–30% (low expression), 38.5% exhibited an IPS of 31–60%, and 19.2% demonstrated an IPS of 61–100%. When a cutoff value of ≥31% was applied to define the high IPS group, 57.7% of patients were classified as having high IPS expression.

### 3.3. Correlation of S100A8 Expression and Clinicopathological Features

Table 2 summarizes the correlations between S100A8 expression patterns and various clinicopathological characteristics in the study cohort. Three distinct expression parameters were evaluated: TPS, TI, and IPS. No statistically significant associations were observed between TPS and any of the examined clinicopathological variables. Similarly, TI did not demonstrate a significant association with clinicopathological parameters. Age, tumor size, invasion depth, histological grade, T stage, and N stage did not demonstrate significant correlations with TPS or TI (all *p* > 0.05). By contrast, the IPS showed two statistically significant associations. A significant correlation was observed with patients’ age (*p* = 0.044), where patients aged ≤ 51 years demonstrated a higher proportion of low IPS expression than older patients. Additionally, histological grade demonstrated a strong association with IPS (*p* = 0.012), with lower-grade tumors (G1) predominantly exhibiting low immune compartment expression, while higher-grade tumors (G2 and G3) showed predominantly high IPS expression. Younger patients aged ≤ 51 years) and those with lower-grade tumors (G1) more frequently demonstrated low IPS, whereas older patients and those with higher-grade tumors (G2 and G3) exhibited a high IPS. These findings suggest that S100A8 expression in the immune compartment, rather than in tumor cells, may serve as a more relevant biomarker associated with the demographic characteristics and tumor differentiation in patients with EC.

### 3.4. Correlation Among TPS, TI, and IPS

Table 3 shows the correlation of S100A8 expression between tumor and immune cells in the study cohort. Among patients with a low TPS, 15 (55.6%) exhibited a low IPS, whereas 12 (44.4%) showed a high IPS. By contrast, among patients with high TPS, 7 (28%) had a low IPS and 18 (72.0%) had a high IPS. A significant positive correlation was identified between TPS and IPS (*p* = 0.044). Tumors with a low TPS frequently demonstrated a correspondingly low IPS, whereas those with a high TPS exhibited an elevated IPS, indicating a parallel expression pattern between tumor and immune cells within the tumor microenvironment. No significant association was observed between TI and IPS (*p* = 0.253), suggesting that the intensity of S100A8 staining in tumor cells did not correlate with the expression levels of S100A8 in immune cells.

## 4. Discussion

The global incidence of endometrial cancer has more than doubled over the past three decades, demonstrating a 2.55-fold increase since 1990 [1]. Endometrioid EC constitutes the majority of cases; however, aggressive subtypes such as serous carcinoma, clear cell carcinoma, and carcinosarcoma disproportionately contribute to morbidity and mortality [4,5]. In South Korea, the incidence of endometrial cancer has steadily increased across all histological subtypes, with survival gains observed primarily in endometrioid carcinoma, while the outcomes for serous and clear cell subtypes have remained largely unchanged [2,3]. Survival gains remain limited in serous and clear cell endometrial carcinomas owing to their aggressive biology, chemoresistance, rarity, and lack of effective targeted therapies [2]. Even within endometrioid carcinoma, prognosis remains heterogeneous. EC is characterized by diverse histopathological and molecular features. Traditional risk stratification relies on histological subtype, tumor grade, and stage; however, these parameters often fail to fully capture the biological aggressiveness of individual tumors [6].

Advances in genomic profiling have led to the development of molecular classification systems, most notably The Cancer Genome Atlas (TCGA), which stratifies EC into four molecular subgroups: POLE-ultramutated tumors with excellent prognosis, MSI-H/MMRd tumors characterized by immune infiltration and generally associated with favorable outcomes, p53-abnormal tumors associated with poor prognosis, and NSMP tumors with intermediate outcomes [24,26,27]. Molecular classification has been incorporated into major clinical guidelines, including those of the ESGO/ESTRO/ESP and NCCN, to refine risk stratification and guide adjuvant treatment decisions for EC [4,5]. However, despite its prognostic value, integration into routine practice is challenging. The limited availability of molecular testing, variability in interpretation, and overlap between histopathological and molecular features complicate individualized treatment. Moreover, survival outcomes have not markedly improved, particularly in the p53abn and NSMP subgroups, underscoring the need for more effective targeted therapies and biomarker-driven approaches. Poor clinical outcomes in endometrial carcinoma are frequently associated with high histological grade, advanced stage, lymphovascular space invasion, and molecular alterations, particularly p53 mutations [5,6]. These adverse factors contribute to increased recurrence rates and reduced survival, even among patients with endometrioid carcinomas, who generally have a more favorable prognosis than those with serous or clear cell carcinomas. Collectively, these findings highlight the heterogeneity of EC and underscore the need to integrate histopathological, stage-related, and molecular features for more precise risk stratification and individualized management [4,5,24]. A subset of patients demonstrates unexpectedly aggressive clinical behavior, emphasizing the urgent need for novel biomarkers that can refine prognostic assessment and guide therapeutic decision-making. In this regard, the absence of reliable biomarkers for early detection and risk stratification remains a major challenge in EC management. Recent evidence suggests that members of the S100 protein family may represent promising candidates.

The S100 protein family, first identified in the bovine brain in 1965 [7], comprises more than 20 members with conserved EF-hand calcium-binding domains and distinct biological functions. These small proteins are predominantly clustered on chromosome 1q21 [8]. They are frequently dysregulated in various malignancies and influence cellular processes such as growth, cytoskeletal regulation, and differentiation, as well as tumor development and inflammation [9]. The S100 protein family has been implicated in tumorigenesis in various malignancies, with S100A8 emerging as a particularly important regulator of tumor–immune interactions [9]. S100A8—a calcium-binding EF-hand protein primarily expressed in myeloid immune cells—plays a key role in immune regulation, inflammation, and various cellular processes such as proliferation, migration, and survival [24,28]. The tumor immune microenvironment is increasingly recognized as a key driver of cancer progression and a potential therapeutic target. MDSCs contribute to immunosuppression, tumor invasion, angiogenesis, and metastasis [12,29]. S100A8 serves as a surrogate marker for MDSCs and promotes their recruitment and accumulation via autocrine signaling [13]. It is involved in tumor proliferation, migration, and premetastatic niche formation [12,14]. Notably, S100A8 is expressed in both immune and tumor cells [14], and its overexpression has been reported in multiple solid tumors and is consistently associated with poor survival [15,16,17,18,19,20]. A meta-analysis data further supported its prognostic significance, showing that high S100A8 expression is associated with worse overall, disease-free, and progression-free survival, particularly in patients with breast and bladder cancers, and was found to be associated with tumor differentiation and lymphatic metastasis [30]. These findings highlight S100A8 as a potential biomarker of cancer progression and prognosis. Mechanistically, S100A8 engages RAGE and TLR4 signaling to activate NF-κB and MAPK pathways, promoting cytokine release and myeloid-derived suppressor cell recruitment.

Multiple S100 proteins, including S100A2, S100A4, S100A8, and S100A9, are significantly upregulated in EC compared with those in the normal endometrium, and their expression has been linked to tumor proliferation, invasion, and progression [11,22,23]. For example, S100A4 promotes EMT and cancer stem cell properties [22], while S100A1 influences cell cycle arrest and apoptosis [23]. S100A8 has been implicated in both tumor growth and chemotherapy sensitivity, and functional studies have shown that its downregulation increases apoptosis and enhances response to paclitaxel [11]. Beyond tumor-intrinsic effects, S100A8 expression in immune cells may influence the tumor microenvironment and correlate with clinical outcomes. Clinically, high expression of S100A1, S100A2, S100A4, and S100A9 is associated with poor survival and has been identified as an independent prognostic factor for EC [11,21,22,23]. These proteins also interact with the tumor immune microenvironment, with S100A8 and S100A9 being associated with immune infiltration and inflammatory pathways [31]. Collectively, these findings suggest that S100 family members may serve not only as prognostic biomarkers but also as potential therapeutic targets for EC. However, the clinicopathological significance of S100A8 in gynecologic malignancies remains poorly understood, and existing studies are limited. Furthermore, an integrated evaluation of S100A8 expression in both the tumor and immune compartments of EC has not yet been reported.

This study provides the first comprehensive evaluation of S100A8 expression in both the tumor and immune compartments of endometrioid EC using TMAs. We observed heterogeneous S100A8 expression patterns, with nearly half of the tumors exhibiting a low TPS, two-thirds showing high TI, and more than half displaying a high IPS. Importantly, S100A8 expression in tumor cells (TPS and TI) was not significantly associated with clinicopathological parameters, suggesting that tumor-intrinsic S100A8 may not directly reflect disease aggressiveness. By contrast, immune-related expression (i.e., IPS) significantly correlated with patients’ age and histological grade, indicating that S100A8 activity in the immune microenvironment may be linked to tumor differentiation and host immune responses. A strong positive correlation between TPS and IPS further supports the possibility of crosstalk between the tumor and immune compartments, consistent with prior evidence implicating S100A8 in the recruitment of MDSCs and modulation of inflammatory pathways. Our findings suggest that immune-associated, rather than tumor-intrinsic, S100A8 expression may hold greater clinical relevance as a biomarker of endometrioid EC. The absence of an association between TI and IPS further emphasizes that the staining intensity in tumor cells alone is insufficient to capture the immunological dynamics of S100A8 expression. Although previous studies on breast and colorectal cancers have associated high tumor S100A8 expression with aggressive features and poor outcomes [15,32], our data suggest that immune-related expression patterns may provide more meaningful insights into EC. The absence of significant associations between tumor-intrinsic S100A8 expression (TPS and TI) and clinicopathological parameters in endometrial carcinoma may reflect its heterogeneous and context-dependent biology. In contrast to other malignancies where tumor-derived S100A8 promotes invasion and poor outcomes, its function in EC appears more immunomodulatory than oncogenic. This divergence likely stems from the unique hormonal and inflammatory microenvironment of the endometrium, where immune cell-derived S100A8 plays a dominant role in shaping tumor–immune crosstalk. The significant association of immune-related S100A8 expression with patient age and histologic grade supports its link to age-related immune dysregulation and tumor dedifferentiation. Increased infiltration of S100A8 myeloid cells may indicate enhanced inflammatory activity and immune remodeling. Collectively, these findings suggest that in EC, S100A8 serves as a biomarker of immune context rather than a direct driver of tumor progression, highlighting the importance of immune–epithelial interactions in endometrial tumor biology.

Several limitations should be noted, including the retrospective design and small sample size, which may reduce sensitivity to subtle associations. Although the cohort (2002–2009) is historical, the biological relevance of S100A8 expression and its correlation with key clinicopathological factors remain valid, and archived tissues continue to provide valuable material for biomarker studies. Validation in modern, molecularly classified cohorts is warranted to confirm clinical relevance. Second, a major limitation is the absence of TCGA-based molecular classification, as our historical cohort was staged according to the 2009 FIGO criteria without POLE, MMR, p53, or NSMP profiling. Given that these molecular subgroups exhibit distinct prognostic and immune profiles, the lack of molecular data limits assessment of potential variation in S100A8 expression across TCGA-defined classes. Future studies incorporating molecular stratification are needed to elucidate the prognostic and immunobiological significance of S100A8 within these molecular subtypes. Third, Survival analysis was not performed due to the predominance of patients with early-stage disease (92.3% stage I–II) and favorable histologic features within our cohort. Consequently, the number of recurrence or mortality events was insufficient for robust statistical modeling, rendering the study underpowered to evaluate survival outcomes. Fourth, functional analyses to clarify the mechanistic role of S100A8 in endometrial cancer progression were not performed. Fifth, S100A8 expression was semi-quantitatively assessed using a three-tier system to enhance reproducibility, though this categorical approach may reduce statistical sensitivity; larger studies using continuous or ROC-based modeling are warranted to refine cutoff values. Future studies incorporating larger cohorts and molecular investigations are needed to validate the clinical utility of S100A8 as a prognostic or predictive biomarker and to further delineate its role in modulating the tumor–immune microenvironment.

## 5. Conclusions

This study provides the first comprehensive assessment of S100A8 expression in both the tumor and immune compartments of endometrioid EC. Our findings indicate that immune cell-associated S100A8 expression, rather than tumor-intrinsic expression, correlates with patients’ age and histological grade, suggesting that it more accurately reflects the tumor–immune microenvironment. The observed positive correlation between tumor and immune cell expression of S100A8 further supports the possibility of crosstalk between these compartments mediated by S100A8. These results highlight the importance of evaluating tumor–immune interactions in biomarker development. Although TI alone may not be sufficient as a prognostic marker, immune compartment expression of S100A8 emerges as a promising candidate for future prognostic and therapeutic applications. Larger prospective studies incorporating functional analyses are warranted to validate these findings and elucidate the mechanistic role of S100A8 in the progression and immune modulation of endometrial cancer.

## Figures and Tables

**Table 1:** Clinicopathological features and S100A8 expression in patients with endometrial carcinoma (*n* = 52).

Variables		Value (Median or Proportion)
Age (years)		35–78 (51)
Tumor size (cm)		0.7–10 (3.5)
Invasion depth (cm)		0.01–4 (0.3)
T stage	1a	36 (69.2%)
	1b	12 (23.1%)
	2	2 (3.8%)
	3a	1 (1.9%)
	3b	1 (1.9%)
N stage	0	47 (90.4%)
	1	3 (5.8%)
	2	2 (3.8%)
FIGO histologic grade	G1	35 (67.3%)
	G2	13 (25.0%)
	G3	4 (7.7%)
S100A8 TPS *	0–30%	27 (51.9%)
	31–60%	16 (30.8%)
	61–100%	9 (17.3%)
S100A8 TI **	Negative	6 (11.5%)
	Weak	13 (25.0%)
	Moderate	21 (40.4%)
	Severe	12 (23.1%)
S100A8 IPS ***	0–30%	22 (42.3%)
	31–60%	20 (38.5%)
	61–100%	10 (19.2%)

TPS: tumor cell Proportion Score; TI: tumor cell staining intensity; IPS: immune cell proportion score. *, percentage of observed tumor cells with confirmed S100A8 cytoplasmic expression; **, value of the most intense tumor cells expressing S100A8; ***, percentage of S100A8-expressing immune cells in the tumor tissue.

**Table 2:** Correlation between S100A8 expression and clinicopathological characteristics (*n* = 52).

Variable		S100A8 TPS			S100A8 TI			S100A8 IPS		
		Low *	High	*p*-Value	Low **	High	*p*-Value	Low ***	High	*p*-Value
Age (years)	≤51	14	13	0.991	11	16	0.513	15	12	0.044
	>51	13	12		8	17		7	18	
Tumor size	≤3.5 cm	12	15	0.262	8	19	0.282	9	18	0.173
	>3.5 cm	15	10		11	14		13	12	
Invasion depth	≤0.3 cm	12	10	0.746	7	15	0.545	12	10	0.126
	>0.3 cm	15	15		12	18		10	20	
Histologic grade	G1	16	19	0.199	14	21	0.457	19	16	0.012
	G2.3	11	6		5	12		3	14	
T stage	≤1a	16	20	0.105	12	24	0.472	18	18	0.092
	>1a	11	5		7	9		4	12	
N stage	≤0	25	22	0.462	17	30	0.610	20	27	0.648
	N1,2	2	3		2	3		2	3	

TPS: Tumor cell proportion score; TI: Tumor cell staining intensity: IPS: Immune cell proportion score. *p*, *p*-value; *, low indicates the 0–30% proportion group, and high indicates the 31–100% proportion group; ** low indicates the negative or weak intensity group, and high indicates the moderate-to-severe intensity group; ***, low indicates the 0–30% proportion group, and high indicates the 31–100% proportion group.

**Table 3:** Correlation of S100A8 expression between tumor and immune cells in the study cohort.

		S100A8 IPS		
		Low ***	High	*p*-Value
S100A8_TPS	Low *	15	12	0.044
	High	7	18	
S100A8 TI	Low **	10	9	0.253
	High	12	21	

TPS: Tumor cell proportion score; TI: Tumor cell staining intensity: IPS: Immune cell proportion score. *p*, *p*-value; *, low indicates the 0–30% proportion group, and high indicates the 31–100% proportion group; ** low indicates the negative or weak intensity group, and high indicates the moderate-to-severe intensity group; ***, low indicates the 0–30% proportion group, and high indicates the 31–100% proportion group.

## Data Availability

The datasets used and/or analyzed in the current study are available from the corresponding author upon reasonable request.
